# Prognostic Value of Venous Thromboembolism Risk Assessment Models in Patients with Severe COVID-19

**DOI:** 10.1055/s-0041-1730293

**Published:** 2021-06-22

**Authors:** Luis H. Paz Rios, Iva Minga, Esther Kwak, Ayman Najib, Ashley Aller, Elizabeth Lees, Victor Macrinici, Kaveh Rezaei Bookani, Amit Pursnani, Joseph Caprini, Alex C. Spyropoulos, Alfonso Tafur

**Affiliations:** 1 Cardiovascular Division, Department of Medicine, NorthShore University Health System, Evanston, Illinois, United States; 2 Department of Medicine, NorthShore University Health System, Evanston, Illinois, United States; 3 Pritzker School of Medicine, University of Chicago, Chicago, Illinois, United States; 4 Department of Medicine, Donald and Barbara Zucker School of Medicine at Hofstra/Northwell Anticoagulation and Clinical Thrombosis Services, Northwell Health at Lenox Hill Hospital, NY, NY, United States

**Keywords:** COVID-19, risk assessment, thrombosis, mortality predictor, IMPROVE score, Caprini score

## Abstract

**Introduction** Severe novel corona virus disease 2019 (COVID-19) causes dysregulation of the coagulation system with arterial and venous thromboembolism (VTE). We hypothesize that validated VTE risk scores would have prognostic ability in this population.

**Methods** Retrospective observational cohort with severe COVID-19 performed in NorthShore University Health System. Patients were >18 years of age and met criteria for inpatient or intensive care unit (ICU) care. The International Medical Prevention Registry on Venous Thromboembolism (IMPROVE) and Caprini scores were calculated and patients were stratified.

**Results** This study includes 184 patients, mostly men (63.6%), Caucasian (54.3%), 63 years old (interquartile range [IQR]: 24–101), and 57.1% of them required ICU care. Twenty-seven (14.7%) thrombotic events occurred: 12 (6.5%) cases of disseminated intravascular coagulation (DIC), 9 (4.9%) of pulmonary embolism, 5 (2.7%) of deep vein thrombosis, and 1 (0.5%) stroke. Among them, 86 patients (46.7%) died, 95 (51.6%) were discharged, and 3 (1.6%) were still hospitalized. “Moderate risk for VTE” and “High risk for VTE” by IMPROVE score had significant mortality association: (hazard ratio [HR]: 5.68; 95% confidence interval [CI]: 2.93–11.03; *p* < 0.001) and (HR = 6.22; 95% CI: 3.04–12.71; *p* < 0.001), respectively, with 87% sensitivity and 63% specificity (area under the curve [AUC] = 0.752, *p* < 0.001). “High Risk for VTE” by Caprini score had significant mortality association (HR = 17.6; 95% CI: 5.56–55.96; *p* < 0.001) with 96% sensitivity and 55% specificity (AUC = 0.843, *p* < 0.001). Both scores were associated with thrombotic events when classified as “High risk for VTE” by IMPROVE (HR = 6.50; 95% CI: 2.72–15.53; *p* < 0.001) and Caprini scores (HR = 11.507; 95% CI: 2.697–49.104; *p* = 0.001).

**Conclusion** The IMPROVE and Caprini risk scores were independent predictors of mortality and thrombotic events in severe COVID-19. With larger validation, this can be useful prognostic information.

## Introduction

Initially described in China, the novel coronavirus disease 2019 (COVID-19) pandemic is caused by the severe acute respiratory syndrome-coronavirus-2 (SARS-CoV-2) 1 and has resulted in significant morbidity and mortality worldwide with approximately 1,600,000 deaths as of December 15, 2020, 2 constituting an urgent threat to global health.

Most affected individuals with COVID-19 will present a milder and self-limited form of the disease with flu-like symptoms. 3 However, a smaller portion will develop severe or critical illness and potentially acute respiratory distress syndrome, systemic inflammatory response syndrome, and multiorgan failure with associated mortality in up to 50% of cases. 4

It is known that hospitalized patients with acute medical illness are at increased risk for the development of venous thromboembolism (VTE). 5 In addition to this, there is a growing body of evidence showing the direct and indirect endothelial damage that occurs as a consequence of COVID-19, further predisposing patients to thrombotic disease in the venous and the arterial circulation. 6 7

As the number of cases of COVID-19 steadily increases, tools with the ability to risk stratify and triage these affected individuals can help allocate resources and optimize medical care. Several risk factors for disease severity and poorer outcomes have been identified 1 8 9 ; however, controversy remains in the optimal way to classify these patients. 10 Considering that the particular endothelial involvement and the high incidence of VTE in patients with COVID-19 are highly associated with worse outcomes, 11 we hypothesized that current validated and readily available VTE risk assessment models (RAM) like the International Medical Prevention Registry on Venous Thromboembolism (IMPROVE) and Caprini scores, would provide prognostic information and predict mortality in patients with severe COVID-19 disease. 12 13

## Methods

### Study Patients

This was a retrospective observational study of consecutive hospitalized patients with severe COVID-19 disease, performed in NorthShore University Health System from March 12, 2020, through July 30, 2020. Patients were at least 18 years of age with SARS-CoV-2 infection confirmed by polymerase chain reaction. Severe COVID-19 disease was defined by the presence of with dyspnea, hypoxia (peripheral saturation < 92%), need for oxygen supplementation, or significant lung involvement on imaging. All patients met criteria for inpatient-level care (IC) or intensive care unit (ICU)-level care when they required invasive ventilatory support.

On April 15, an institutional thromboprophylaxis protocol for patients with COVID-19 was implemented in line with multisociety recommendations. 14 All patients underwent individualized VTE risk stratification with the IMPROVE score or Caprini score. Thromboprophylaxis with low molecular weight heparin (LMWH) at prophylactic or intermediate doses (i.e., enoxaparin 40 mg sub cutaneous daily or 40 mg s.c. twice daily if patients met criteria: body mass index [BMI] >30 kg/m ^2^ , need for high-flow nasal cannula, mechanical ventilation, sepsis-induced coagulopathy score of ≥4, and D-dimer > 6 times upper limit of normal) was the preferred treatment modality, unless patients had severe renal insufficiency. Furthermore, thromboprophylaxis was extended for 2 weeks with LMWH for patients over 40 years of age with reevaluation of VTE risk at follow-up. Alternatively, extended thromboprophylaxis up to 45 days with rivaroxaban 10 mg daily was recommended when criteria were met (over 50 years old, IMPROVE VTE risk ≥ 4, Caprini score >8, D-dimer > 1 mg/dL, ICU stay, prior VTE, active cancer, and paralysis).

The present study was approved by the local Institutional Review Board (Federal Wide Assurance: FWA00003000) and written consent was waived given its retrospective design. The database with deidentified patient information was set up and maintained by the Cardiovascular Division. 15

### Data Collection

Faculties of the Department of Internal Medicine and the Division of Cardiology settled and extracted data by review of the institutional electronic medical record. We abstracted demographic data including age, gender, BMI, and laboratory parameters at hospitalization. Comorbidities, defined as simultaneous presence of >1 chronic condition, were recorded on hospital admission.

Laboratory data were collected within the first 5 days from hospitalization and, taking into consideration, the available peak values.

The IMPROVE and Caprini scores were calculated at the time of data collection and by a second author at the time of data analysis for corroboration. Patients were classified according to the IMPROVE risk score in “Low risk for VTE” (score 0–1), “Moderate risk for VTE” (score 2–3), and “High risk for VTE” (score ≥ 4). 16 17

For the Caprini score, patients were classified according to their risk scores and dichotomized into “Low to Moderate risk for VTE” (score 0–4) and “High Risk for VTE” (score ≥ 5) to make groups comparable, and were subsequently stratified by Caprini score at accepted cut-offs as follows: very low risk (Caprini score: 0–2), moderate risk (Caprini score: 3–4), high risk (Caprini score 5–6), very high risk (Caprini score: 7–8), and the highest risk (Caprini score >8). 18 19

### Outcomes

VTE was defined as deep vein thrombosis (DVT) or pulmonary embolism (PE) when clinically observed and treated during hospitalization. Patients were considered to have DVT if treated for DVT and/or had positive compression ultrasonography test. Routine screening for DVT was not instituted by the time of adjudication, this was in line with the recommendations from the World Health Organization until the time of censoring. Patients were considered to have PE if they were treated for PE and had a positive lung scan, pulmonary angiogram, or spiral computed tomography (CT) scan. The International Society for Thrombosis and Hemostasis (ISTH) criteria for the diagnosis of disseminated intravascular coagulation (DIC) was used.

Clinical and mortality outcomes were extracted and censored at the time of death and at time of last follow-up until July 30, 2020. The data and scores were verified by a second independent author for their accuracy and, in case of discrepancy, adjusted by consensus.

### Statistical Analysis

Descriptive data were summarized to characterize the distribution of baseline parameters. Kolmogorov–Smirnov and Shapiro–Wilk tests were used to evaluate if continuous variables were normally distributed; we used Student's *t* -test to compare normally distributed continuous variables and Mann–Whitney *U* -test to compare nonparametric continuous variables; Pearson's Chi-square test or Fisher's exact test were used to compare categorical variables.

We performed univariate and multivariate analyses using Cox's proportional hazards regression with forward modeling to explore the association of each variable and mortality, as well as thrombotic outcomes. Variables with statistical significance in univariate analysis ( *p* < 0.05) were included in multivariate analysis to identify independent risk factors for mortality and thrombotic outcomes. Development of a thrombotic outcomes was treated as a time-dependent variable to eliminate the effect of misclassification from immortal time bias.

We used the receiver-operating characteristics (ROCs) curve to analyze the discriminatory capacity of each RAM, and used the Kaplan–Meier method to estimate the cumulative event-free survival curves for the different groups.

We present categorical variables as absolute numbers and percentages, and continuous variables by their mean and standard deviation, or by the median and interquartile range (IQR) as appropriate. All statistical analyses were performed with SPSS version 27.0 (IBM, Armonk, New York, United States). A *p* -value of <0.05 was considered statistically significant.

## Results

A total of 184 consecutive patients with severe COVID-19 were included for analysis. The patients in this study were predominantly men (63.6%), Caucasian (54.3%), 63 years of age (IQR: 24–101 years), and the majority (57.1%) required ICU-level care. Patients were hospitalized on average 1 day (IQR: 0–25 days) after diagnosis of COVID-19, spent on average 7 days (IQR: 0–68 days) in the hospital, and were followed-up for a median of 52 days (IQR: 0–108 days) from their initial encounter.

At the time of censoring, a total of 27 (14.7%) thrombotic events had occurred. There were 12 (6.5%) cases of DIC, 9 (4.9%) cases of pulmonary embolism, 5 (2.7%) DVT, and 1 (0.5%) stroke. A total of 86 patients (46.7%) died, 95 patients (51.6%) were discharged, and 3 patients (1.6%) were still hospitalized and undergoing medical care at the time of censoring.

Comparison between the groups showed that mortality was significantly higher in patients who were older, had hypertension, diabetes mellitus, coronary artery disease, cerebrovascular disease, cancer, and patients who required ICU-level care. Mortality was significantly higher in those classified as “Moderate risk for VTE” and “High risk for VTE” by the IMPROVE score, and “High risk for VTE” by the Caprini score ( Table 1 ).

**Table 1:** Epidemiologic characteristics of critically ill patients with COVID-19, NorthShore University Health System

Variables	Cohort ( *n* = 184)		Alive ( *n* = 98)		Deceased ( *n* = 86)		c *p* -Value
	Mean/median/ *n*	SD/IQR/%	Mean/median/ *n*	SD/IQR/%	Mean/median/ *n*	SD/IQR/%	
a Age (y)	63	24–101	59	24–101	67.5	37–91	0.003
b Age ≥ 65 (y)	85	46.2	34	34.7	51	59.3	<0.001
b Gender							
Female	67	36.4	34	34.7	33	38.4	0.605
Male	117	63.6	64	65.3	53	61.6	
a BMI (kg/m ^2^ )	30.4	8.0	29.8	7.2	31.1	8.8	0.271
b Ethnicity							
Caucasian	100	54.3	52	53.1	48	55.8	0.495
Black	12	6.5	5	5.1	7	8.1	
Hispanic	48	26.1	30	30.6	18	20.9	
Asian	18	9.8	9	9.2	9	10.5	
Other	6	3.3	2	2.0	4	4.7	
b Type of residence							
Private home	148	80.4	82	83.7	66	76.7	0.214
Nursing home	32	17.4	13	13.3	19	22.1	
Other	4	2.2	3	3.1	1	1.2	
b Social history							
Smoking history	56	30.4	24	24.5	32	37.2	0.061
Alcohol use	58	31.5	34	34.7	24	27.9	0.278
Recreational drugs	3	1.6	1	1.0	2	2.3	0.497
a Hospitalization length (d)	7	0–68	6	0–68	9	0–63	0.439
b Level of care							
Inpatient care	79	42.9	67	68.4	12	14.0	<0.001
Intensive care unit	105	57.1	31	31.6	74	86.0	
b Comorbidities							
Hypertension	99	53.8	40	40.8	59	68.6	<0.001
Hyperlipidemia	71	38.6	32	32.7	39	45.3	0.078
Diabetes	75	40.8	29	29.6	46	53.5	0.001
Coronary disease	21	11.4	6	6.1	15	17.4	0.016
Heart failure	18	9.8	6	6.1	12	14.0	0.074
CVA	12	6.5	3	3.1	9	10.5	0.042
CKD	19	10.3	8	8.2	11	12.8	0.303
Atrial fibrillation	23	12.5	8	8.2	15	17.4	0.058
COPD	15	8.2	6	6.1	9	10.5	0.283
Cancer	20	10.9	6	6.1	14	16.3	0.027
VTE	9	4.9	4	4.1	5	5.8	0.587
b VTE risk assessment models							
Caprini high risk	128	69.6	45	45.9	83	96.5	<0.001
IMPROVE moderate VTE risk	39	21.2	13	13.3	26	30.2	<0.001
IMPROVE high VTE risk	72	39.1	23	23.5	49	57.0	<0.001
b Thrombotic events							
DVT	5	2.7	3	3.1	2	2.3	0.067
Pulmonary embolism	9	4.9	4	4.1	5	5.8	
DIC	12	6.5	3	3.1	9	10.5	
Stroke	1	0.5	0	0	1	1.2	

Abbreviations: BMI, body mass index; CKD, chronic kidney disease; COPD, chronic obstructive pulmonary disease; COVID-19, novel coronavirus disease 2019; CVA, cerebrovascular accident; DIC, disseminated intravascular coagulation; DVT, deep vein thrombosis; IMPROVE, the International Medical Prevention Registry on Venous Thromboembolism; IQR, interquartile range; SD, standard deviation; VTE, venous thromboembolism.

a Normally distributed continuous variables, presented as mean with standard deviation or median and interquartile range.

b Categorical variables, presented as number and percentage of patients.

c *p* -Value obtained with Student's *t* -test for continuous variables, Chi-square test or Fisher's exact test for categorical variables.

Direct comparison of laboratory values collected revealed that nonsurvivors had significantly lower hemoglobin and albumin levels. Also, nonsurvivors had significantly higher prothrombin time, international normalized ratio (INR), D-dimer levels, creatinine levels, C-reactive protein levels, aspartate transaminase, and significantly higher absolute lymphocyte count ( Table 2 ).

**Table 2:** Laboratory values during hospitalization for COVID-19 critically ill patients, NorthShore University Health System

Variables	Cohort	Alive	Deceased	c *p* -Value
b Hgb (gr/dL)	12.2	12.9	11.5	<0.001
b Neutrophil count (10 ^9^ /L)	8.2	7.8	8.7	0.087
b Lymphocyte count (10 ^9^ /L)	1.2	1.0	1.6	0.035
b Platelet count (10 ^9^ /L)	231.3	240.5	220.9	0.206
b Fibrinogen (mg/dL)	683.1	704.6	669.3	0.950
b PT (s)	14.5	13.0	15.9	<0.001
b INR	1.4	1.2	1.6	<0.001
b D-dimer (mg/L)	7.8	5.3	9.7	<0.001
b Creatinine level (mg/dL)	1.6	1.0	2.2	<0.001
b Ferritin (ng/mL)	2454	1489	2951	0.570
b C-reactive protein (mg/dL)	184.2	146.7	225.3	<0.001
b LDH (U/L)	567.2	479.7	607.9	0.114
b AST (U/L)	186.2	52.2	283.3	0.002
b ALT (U/L)	74.4	47.8	94.5	0.886
a Albumin (mg/dL)	2.3	2.7	2.0	<0.001

Abbreviations: ALT, alanine transaminase; AST, aspartate transaminase; Hgb, hemoglobin; INR, international normalized ratio; LDH, lactate dehydrogenase; PT, prothrombin time.

a Parametric continuous variable.

b Nonparametric continuous variable.

c *p* -Values obtained with Student's *t* -test for parametric continuous variables; Wilcoxon's rank-sum test and Mann-Whitney *U* -test were used for nonparametric continuous variables.

### The IMPROVE Score and Mortality

**Fig. 1:**
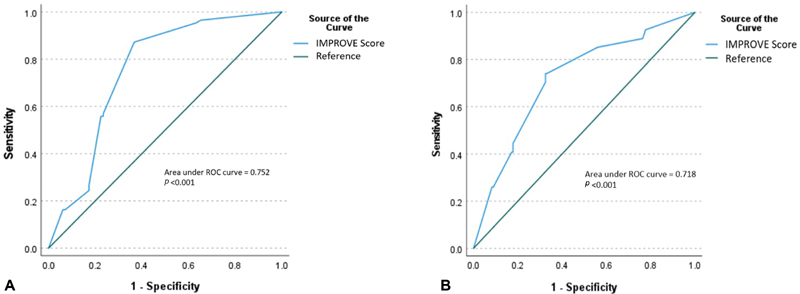
( **A** ) Receiver-operating characteristics (ROC) curve of IMPROVE score for prediction of mortality. ( **B** ) Receiver-operating characteristics curve of IMPROVE score for prediction of thrombotic events. IMPROVE, the International Medical Prevention Registry on Venous Thromboembolism.

**Fig. 2:**
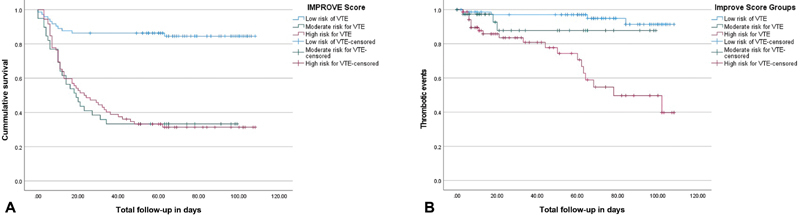
( **A** ) Kaplan–Meier curve demonstrating survival of different groups by IMPROVE score. ( **B** ) Kaplan-Meier curve demonstrating cumulative thrombotic event survival of different groups by IMPROVE score. IMPROVE, the International Medical Prevention Registry on Venous Thromboembolism; VTE, venous thromboembolism.

In univariate analysis, patients classified by the IMPROVE score as “Moderate risk for VTE” and “High risk for VTE” had a significant association with mortality with univariate hazard ratio (HR) = 2.02; 95% confidence interval (CI): 1.27–3.21; *p* = 0.003, and HR = 2.49; 95% CI: 1.62–3.83; *p* < 0.001, respectively. When adjusted for other variables in multivariate Cox's regression analysis, the association remained significant for “High risk for VTE” (HR = 6.22; 95% CI: 3.04–12.71; *p* < 0.001), followed by “Moderate risk for VTE” (HR = 5.68; 95% CI: 2.93–11.03; *p* < 0.001), the presence of diabetes (HR = 1.70; 95% CI: 1.10–2.63; *p* = 0.016), and hypertension (HR = 1.63; 95% CI: 1.02–2.59; *p* < 0.040). An ROC curve was plotted with an area under the curve (AUC) of 0.752 ( *p* < 0.001) and a corresponding sensitivity of 87% and specificity of 63% for the patients classified as “Moderate risk for VTE” or above ( Fig. 1A ). When stratified by IMPROVE scores, the cumulative mortality increased in a linear fashion with increasing score. Patients classified at “Low risk for VTE” had an in-hospital mortality of 15.1%, “Moderate risk for VTE” had a mortality of 66.7%, and “High risk for VTE” had a mortality of 68.1% ( Fig. 2A ).

### Caprini Risk Assessment Model and Mortality

**Fig. 3:**
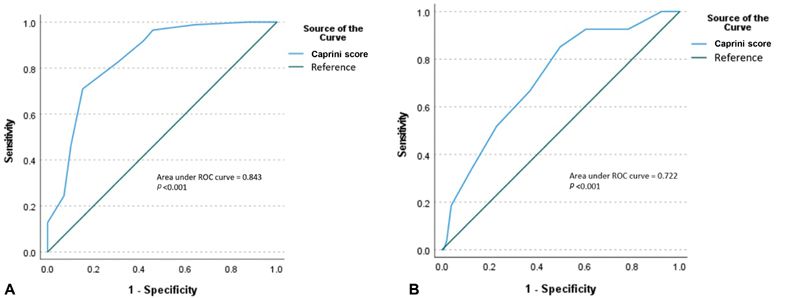
( **A** ) Receiver-operating characteristics curve (ROC) of Caprini score for prediction of mortality. ( **B** ) Receiver-operating characteristics curve of Caprini score for prediction of thrombotic events.

In univariate analysis, patients classified by the Caprini RAM as “High Risk for VTE” had a significant association with mortality (HR = 18.6; 95% CI: 5.87–59.06; *p* < 0.001). Furthermore, this association remained significant when adjusted in multivariate Cox's regression analysis (HR = 17.6; 95% CI: 5.56–55.96; *p* < 0.001), followed by the presence of diabetes (HR = 1.60; 95% CI: 1.05–2.46; *p* = 0.029). An ROC curve was plotted with an AUC of 0.843 ( *p* < 0.001) and a corresponding sensitivity of 96% and specificity of 55% for the designated dichotomized cut-off ( Fig. 3A ).

**Fig. 4:**
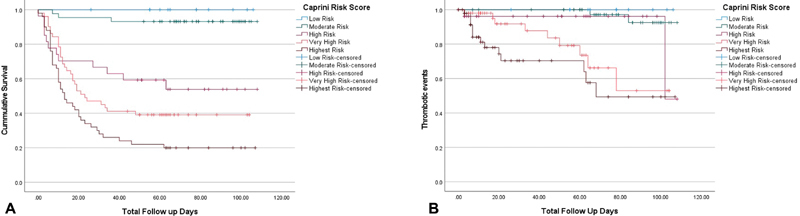
( **A** ) Kaplan–Meier curve demonstrating survival of different groups by Caprini score. ( **B** ) Kaplan–Meier curve demonstrating cumulative thrombotic event survival of different groups by Caprini score.

When stratified by Caprini scores, the cumulative mortality increased in a linear fashion with increasing score. Patients classified at very low risk (Caprini score: 0–2) had no mortality events. Those stratified at moderate risk (Caprini score: 3–4) had 6.8% mortality, high risk (Caprini score: 5–6) had a 44.4% mortality, very high risk (Caprini score: 7–8) had a 60.8% mortality, and the highest risk (Caprini score > 8) had a mortality rate of 80% ( Fig. 4A ).

### Venous Thromboembolism Risk Assessment Models in Prediction of Thrombotic Events

In univariate and multivariate analyses, both scores were statistically associated with thrombotic event occurrence when classified as “High risk for VTE” by IMPROVE score (HR = 6.50; 95% CI: 2.72–15.53; *p* < 0.001) and “High risk for VTE” by Caprini score (HR = 11.507; 95% CI: 2.697–49.104; *p* = 0.001). Patients, classified as “Moderate risk for VTE” by IMPROVE score, did not statistically associate with thrombotic event occurrence (HR = 2.82; 95% CI: 0.62–12.75; *p* = 0.177)

The ROC curve for thrombotic event prediction with the IMPROVE score had an AUC of 0.718 ( *p* < 0.001) with corresponding 85% sensitivity and 44% specificity for those classified as “High risk for VTE” ( Fig. 1B ). The cumulative incidence of VTE for those at “Low risk for VTE” was 5.5%, “Moderate risk for VTE” was 7.7%, and “High risk for VTE” was 27.8% ( Fig. 2B ).

The ROC curve for thrombotic event prediction with the Caprini score had an AUC of 0.722 ( *p* < 0.001) with corresponding 92% sensitivity and 35% specificity for the designated dichotomized cut-off ( Fig. 3B ). When stratified by Caprini scores, the cumulative incidence of VTE increased in a linear fashion with increasing score. Patients classified at very low risk (Caprini score: 0–2) had no VTE events. Those stratified at moderate risk (Caprini score: 3–4) had a VTE incidence of 4.5%, high risk (Caprini score: 5–6) had a VTE incidence of 7.4%, very high risk (Caprini score 7–8) had a VTE incidence of 17.6%, and the highest risk (Caprini score >8) had a VTE incidence of 28.0% ( Fig. 4B ).

## Discussion

In our cohort of hospitalized patients with severe COVID-19 disease, the IMPROVE and Caprini risk assessment models were both strong and independent predictors of mortality. Similarly, both scores were strong and independent predictors of thrombotic event occurrence in those classified as “High risk for VTE” by either RAM. The high mortality seen in our patients with severe COVID-19 disease is similar to prior reports. 4

Severe COVID-19 is associated with dysregulation of the coagulation system, high incidence of arterial and venous thrombosis in large vessels and microvascular beds, and associated poor prognosis. 20 This in part, is thought to be a consequence of the dysregulated immune response leading to endothelial dysfunction, increased vascular permeability, and intrinsic thrombophilia. 21 With this in mind, we chose to evaluate the performance of two well-validated VTE scores and challenged their predictive capacity for the hard outcome of mortality in multivariate analysis. We found that patients classified as “Moderate or High risk for VTE” by the IMPROVE RAM or “High risk for VTE” by the Caprini RAM, had significantly higher mortality. Similarly, patients classified as “High risk for VTE” by the IMPROVE score and “High risk for VTE” by Caprini score had a significantly higher number of thrombotic events.

The Caprini risk score is a widely validated RAM that weights independent risk factors for the individual summing up a total score that correlates with the VTE risk. Importantly, it uses data readily available from the patient's history and physical examination without the inclusion of laboratory or imaging data. 22 The IMPROVE VTE RAM is a widely accepted and validated score calibrated in acutely ill medical patients. It encompasses seven independent VTE risk factors in a hospital setting and does not require additional imaging or laboratory information to be completed. 16 In the setting of a global pandemic, placing health care systems under significant constraint, the application of a clinical score that uses readily obtainable data can be a practical strategy for triaging individuals. If limited availability of medical personnel is a limiting factor, patient friendly forms are available in multiple languages and have an excellent correlation with physician-driven forms. 23 24

As the COVID-19 pandemic continues to spread around the globe, there is a better understanding of its clinical and epidemiological behavior. Although most patients will have a mild form of the disease, there is a nonnegligible 5% of patients who progress into severe illness, with reported mortality ranging from 49 to 61.5%. 4 25 In our cohort of patients with severe disease, mortality was comparable (46.7%) to the reported literature. Similarly, the comparison of comorbidities between deceased and survivors revealed significantly older age, and the presence of cardiovascular risk factors and malignancy in those who died, as previously reported. 9 26 27

One of the challenges that continue to burden health care systems around the globe is the adequate allocation of resources when a large number of patients is in need in a given time. To alleviate this burden, efficient diagnosis and resource utilization are prioritized, and reliable prognostic information as provided by RAM could be a determinant for behavioral change.

Several risk-scoring models have been developed and a few internally validated to help prioritize high-risk individuals with COVID-19. 28 29 30 31 32 All these scores rely on clinical data, laboratory data, and imaging features to generate their prediction. It is important to note that at the time of our submission, these original documents were not peer-reviewed nor have undergone the rigors of external validation, and optimization could have occurred before their publication. Furthermore, in a systematic review of the existing prediction models for diagnosis and prognosis of COVID-19, Wynants et al and the COVID-PRECISE (Precise Risk Estimation to optimise covid-19 Care for Infected or Suspected patients in diverse settings) group rendered the existing models at high risk of bias after independent data extraction using the Critical Appraisal and Data Extraction for Systematic Reviews of Prediction Modeling Studies (CHARMS) checklist and prediction model risk of bias assessment tool (PROBAST). 10

Following hospitalization for medical illness, extended thromboprophylaxis (ET) with LMWH, or direct oral anticoagulants (DOACs) can reduce the risk of VTE at the cost of increased bleeding, with net clinical benefit seen by the DOAC rivaroxaban in key low bleed risk subgroups of medically ill patients. 17 33 34 35 36 Currently, the decision to proceed with ET is largely based on individualized risk stratification for VTE and balancing the patients' bleeding risk. This practice is advised in patients with COVID-19 by the current consensus. 37

## Strengths and Limitations

Our analysis showed the strong predictive ability of both VTE risk assessment models for the hard outcome of mortality in this cohort of patients with severe COVID-19. It also validates the predicting capacity for thrombotic events in this population at increased risk. Because routine screening for VTE or autopsies was not performed routinely amid the pandemic, we could not verify if deaths were VTE related. Also, given the initial scarcity of personal protective equipment that was seen nationwide, is likely that the true number of thrombotic events was underestimated.

Despite the strengths of our findings, there are limitations intrinsic to the observational design of our study and the relatively small sample size from a single-center experience conferring a degree of ascertainment bias. Furthermore, the severity of disease from COVID-19 in our cohort introduces selection bias, thus the results may not be generalizable to all comers with COVID-19 disease.

Like many other retrospective studies, we did not perform a formal power calculation. Therefore, our study might be underpowered to draw firm conclusions about the association of the VTE risk tools, mortality, and VTE. Hence, external validation with large prospective cohorts should be pursued to confirm our findings.

Nonetheless, the implementation of either VTE RAM may be a valuable source with prognostic value for the outcome of death or thrombosis. With further validation, this strategy can assist in triaging individuals with severe COVID-19 disease and optimize resource allocation.

## Conclusion

The IMPROVE and Caprini VTE risk assessment models were independent predictors of mortality and thrombotic events in our cohort of patients with severe COVID-19. With largescale validation, this prognostic information can assist triaging individuals and optimize resource allocation.
